# Complete Genome Sequence of Pseudomonas otitidis Strain MrB4, Isolated from Lake Biwa in Japan

**DOI:** 10.1128/MRA.00148-20

**Published:** 2020-04-16

**Authors:** Kentaro Miyazaki, Erina Hase, Tomomasa Maruya

**Affiliations:** a Department of Life Science and Biotechnology, Bioproduction Research Institute, National Institute of Advanced Industrial Science and Technology (AIST), Tsukuba, Ibaraki, Japan; b Department of Computational Biology and Medical Sciences, The University of Tokyo, Kashiwa, Chiba, Japan; c Department of Engineering Science, Graduate School of Informatics and Engineering, The University of Electro-Communications, Chofu, Tokyo, Japan; Loyola University Chicago

## Abstract

We isolated Pseudomonas otitidis strain MrB4 from the near-shore area of Lake Biwa in Japan and generated its complete genome sequence. MrB4 possesses a single circular chromosome of 6,089,454 bp, with ∼97% average nucleotide identity to the P. otitidis type strain MCC10330 (draft genome).

## ANNOUNCEMENT

Pseudomonas otitidis is a Gram-negative bacterium that was initially isolated from clinical specimens of patients with ear infection (1). This species was found to be closely related to P. aeruginosa, but since the 16S rRNA gene sequence of the P. otitidis type strain MCC10330 showed only 98.6% sequence identity to P. aeruginosa (1), P. otitidis was placed at a distinct taxonomical position from P. aeruginosa. Although this species was first discovered in human patients in 2006 (1), it has been reported that P. otitidis is also widespread in nonclinical environments, including desiccation lagoons (2), activated sludge (3, 4), drinking water (5), and animal meat (6). Researchers are interested in P. otitidis for its possession of a unique subclass B3 metallo-beta-lactamase, POM, which was named after Pseudomonas otitidis metallo-beta-lactamase (7, 8).

We collected a water sample from the near-shore area of Lake Biwa, the largest freshwater lake in Japan, on 12 August 2019. The water sample was spread over multiple LB (1% [wt/vol] tryptone, 0.5% [wt/vol] yeast extract, 0.5% [wt/vol] NaCl) agar (1.5% [wt/vol]) plates, and the plates were incubated at 37°C overnight. Several well-separated single colonies were isolated and subjected to DNA sequencing of the near-full-length sequence of the 16S rRNA gene using a set of primers, Bac8f(C) and UN1541r(U) (9). The sequence analysis of 16S rRNA indicated that many of the isolated bacterial colonies were affiliated with P. otitidis. Out of all the isolated strains, one strain, designated MrB4, exhibited 99.7% identity to the 16S rRNA gene sequence of P. otitidis MCC10330^T^ (GenBank accession number NR_043289). Strain MrB4 was then subjected to whole-genome sequencing analysis.

P. otitidis strain MrB4 was allowed to grow in LB broth at 37°C for 18 h. Genomic DNA was extracted following a procedure that was described previously (10). We performed whole-genome sequencing of this bacterium using a hybrid approach, which involved a combination of GridION (Oxford Nanopore Technologies [ONT], UK) and MiSeq (Illumina) sequencing technologies. Sequence analyses were conducted using the default parameter settings of the software throughout this study.

For long-read sequencing, genomic DNA (1 μg) was treated with the Short Read Eliminator XS kit (Circulomics). With the resulting DNA, a library was constructed using the ligation sequencing kit (ONT), and then the sequences were analyzed on a FLO-MIN106 R9.41 flow cell (ONT) for 10 h. Base calling was performed using Guppy v.3.0.3, and as a result, 434,489 reads (1,097 Mb) were generated, with an average length of 2,525.2 bases. The raw reads were quality (Q) filtered (Q, ≧10; read length, ≧1,000 bases) using NanoFilt v.2.3.0 (11). The longest read was 73,512 bases long.

For short-read sequencing, the Nextera DNA Flex library prep kit (Illumina) was used to generate paired-end libraries with approximately 350-bp inserts. Sequencing was performed using the MiSeq reagent kit v.2 (300-cycles) to obtain reads that were 156 bp long. Adapter sequences and low-quality reads were trimmed (Q, ≧30; read length, ≧10 bases) using fastp v.0.20.0 (12), yielding 771,952 paired-end reads, spanning 232 Mb with an average length of 151 bp.

The trimmed sequences of the long and short reads were assembled using Unicycler v.0.4.8 (13) and polished with Pilon v.1.23 (14), resulting in the generation of a single circular chromosome (6,089,454 bp; G+C content, 37.8%). The obtained sequences were submitted to a Web-based annotation pipeline, DFAST v.1.2.4 (15), for automated annotation. The chromosome was found to possess 5,595 coding sequences, 74 tRNA genes, and 12 rRNA genes. To date (as of 13 February 2020), there are two draft genome sequences of P. otitidis strains publicly available. The average nucleotide identity (ANI) of the newly assembled genome was analyzed using the JSpeciesWS online service (16), which revealed that strain MrB4 exhibits 97 and 98% ANI to strains MCC10330^T^ (GenBank accession number NZ_FOJP00000000) (1) and PAM-1 (NZ_PXJI00000000), respectively. This result suggests that all these strains of P. otitidis are closely related to each other irrespective of their distinct habitats (MCC10330^T^ and PAM-1 were both isolated from infected human ear). P. otitidis strain MrB4 is also found to possess a gene coding for POM, and the deduced amino acid sequence (286 amino acids) exhibits >99% identity to the same protein identified in the previously documented P. otitidis strains. Our study provides an essential basis for performing detailed comparative analysis of P. otitidis genomes in future studies.

### Data availability.

The complete genome sequence of P. otitidis MrB4 is available from DDBJ/EMBL/GenBank under accession number AP022642. The raw sequencing data were deposited in the DDBJ SRA database under accession number DRA009573 (BioProject PRJDB9285, BioSample SAMD00204525).
